# Secukinumab provides rapid and sustained pain relief in psoriatic arthritis over 2 years: results from the FUTURE 2 study

**DOI:** 10.1186/s13075-018-1610-3

**Published:** 2018-06-07

**Authors:** Iain B. McInnes, Philip J. Mease, Georg Schett, Bruce Kirkham, Vibeke Strand, Nicole Williams, Todd Fox, Luminita Pricop, Steffen M. Jugl, Kunal K. Gandhi, on behalf of the FUTURE 2 Study Group

**Affiliations:** 1 Institute of Infection, Immunity & Inflammation, College of Medical, Veterinary and Life Sciences, University of Glasgow, 120 University Place, Glasgow, G12 8TA UK; 2 Swedish Medical Center and University of Washington, Seattle, WA USA; 3 Friedrich-Alexander University of Erlangen-Nuremberg and Universitatsklinikum Erlangen, Erlangen, Germany; 4 Guy’s & St Thomas’ NHS Foundation Trust, London, UK; 5 Stanford University School of Medicine, Palo Alto, CA USA; 6 RTI Health Solutions, Durham, NC USA; 7 Novartis Pharma AG, Basel, Switzerland; 8 Novartis Pharmaceuticals Corporation, East Hanover, NJ USA

**Keywords:** Pain relief, Psoriatic arthritis, Secukinumab

## Abstract

**Background:**

Pain is one of the most important domains affecting health-related quality of life (HRQoL) in patients with psoriatic arthritis (PsA). Secukinumab has demonstrated rapid and sustained improvements in signs and symptoms, including HRQoL, among patients with active PsA. This analysis evaluates the effect of secukinumab on patient-reported pain in PsA through 104 weeks of treatment.

**Methods:**

Pain was assessed through week 104 using clinically relevant measures, including change from baseline in a pain visual analog scale (VAS) and Short Form-36 (SF-36) bodily domain scores; proportion of patients reporting improvements equal to or better than minimum clinically meaningful differences in the pain VAS and SF-36 bodily pain domain scores; and proportion of patients with no, moderate, or extreme pain/discomfort measured by the EuroQoL 5-Dimension 3-Level Questionnaire (EQ-5D-3 L) pain item scores. Correlations of pain measures were analyzed using Pearson’s correlation coefficient. Pre-specified analyses of TNF-naïve patients and patients who stopped TNF-inhibitors (TNFis) due to inadequate responses or safety/tolerability (TNF-IR patients) were performed using “as-observed data.”

**Results:**

Mean improvements from baseline in pain VAS scores were greater with secukinumab versus placebo by week 3 (− 16.9; *P* < 0.0001 with secukinumab 300 mg and − 12.6; *P* < 0.05 with secukinumab 150 mg) and sustained through week 104. SF-36 bodily pain domain scores were significantly greater with 300 mg secukinumab and secukinumab 150 mg versus placebo by week 4 (16.2 and 16.3, respectively; *P* < 0.0001 for both), and these changes were maintained through week 104. With both secukinumab 300 mg and secukinumab 150 mg, improvements equal to or better than the minimum clinically meaningful differences in pain VAS and SF-36 bodily pain were significant versus placebo at week 3 and week 4, respectively. At week 4, 15%, 9%, and 5% of patients receiving secukinumab 300 mg, secukinumab 150 mg, and placebo, respectively, reported “no pain/discomfort” measured by EQ-5D-3 L; these proportions increased to week 104 with both secukinumab doses. Similarly, improvements in pain measures were significant in both TNF-naïve and TNF-IR patients.

**Conclusion:**

Secukinumab provided rapid and sustained pain relief in PsA over 2 years of treatment. Improvements in pain were reported regardless of prior exposure to TNFis.

**Trial registration:**

ClinicalTrials.gov, NCT01752634. Registered on 19 December 2012.

**Electronic supplementary material:**

The online version of this article (10.1186/s13075-018-1610-3) contains supplementary material, which is available to authorized users.

## Background

Psoriatic arthritis (PsA) is a chronic progressive, clinically heterogeneous inflammatory arthritis that can manifest as peripheral arthritis, axial disease, enthesitis, dactylitis, and psoriasis of the skin/nails [1, 2]. The disease burden of PsA, taking into account patient-reported outcomes and disease activity measures, is similar to the burden of rheumatoid arthritis and axial spondyloarthritis [3]. A recent analysis from the population-based Multinational Assessment of Psoriasis and Psoriatic Arthritis (MAPP) found that 88% of patients questioned had ongoing joint pain or soreness, and 60% reported > 4 affected joints [4]. The most common locations for joint pain were the knee (41%) followed by finger (26%), hip (19%), ankle (19%), back (18%), and wrist (16%) [4].

The combination of ongoing pain, fatigue, physical impairment, and anxiety likely influence the impact of PsA and reduce health-related quality of life (HRQoL) [5]. Specifically, patients with PsA have impaired HRQoL characterized by poor physical functioning and engagement in daily activities, and pain [6]. In a European League Against Rheumatism (EULAR) initiative to develop and validate the Psoriatic Arthritis Impact of Disease questionnaire (PsAID), patients with PsA identified pain as the most important health domain affecting HRQoL [7]. Additionally, arthritis or joint pain causes a significant economic impact that results in US$6773 greater costs compared to individuals without either [8]. Pain poses an important clinical challenge in the treatment of PsA. In a population-based survey, rheumatologists and dermatologists considered joint pain or swelling to be the critical factor contributing to the severity of PsA [9]. Treatment criteria, scoring indices, and clinical trial domains further reflect the importance of pain in PsA. The Group for Research and Assessment of Psoriasis and Psoriatic Arthritis (GRAPPA) includes pain as an important factor to examine when treating patients with PsA [10]. A patient pain visual analog scale (VAS) score ≤ 15 is one of seven critical criteria for determining minimal disease activity in PsA [11]. Finally, at the Outcome Measures in Rheumatology (OMERACT) 2016 conference, pain was endorsed as part of a core set of domains recommended for study in randomized controlled trials (RCTs) and longitudinal observational studies (LOS) in PsA [12].

Secukinumab, a fully human monoclonal antibody (mAb) that selectively neutralizes interleukin (IL)-17A, has been shown to have significant efficacy in the treatment of moderate-to-severe psoriasis and active PsA, demonstrating rapid onset of action and sustained responses with a consistent safety profile [13, 14]. Since 2015 in the European Union and 2016 in the USA, secukinumab 300 mg and 150 mg have been approved for the treatment of active PsA. In the phase-3 FUTURE 2 study, secukinumab was effective in treating patients not previously exposed (TNF-naïve patients) to TNF-α inhibitors (TNFis) and patients who stopped using up to three previous TNFis due to inadequate responses or for safety/tolerability reasons (TNF-IR patients) [15]. Over 2 years, secukinumab was shown to exhibit rapid and sustained improvements in the signs and symptoms of active PsA, to inhibit radiographic progression, and to improve HRQoL [16, 17]. This post-hoc analysis of FUTURE 2 examines the effect of secukinumab on patient-reported pain in PsA over 104 weeks.

## Methods

Details of FUTURE 2 (NCT01752634), an ongoing, multicenter RCT, have been reported previously [15] and will be briefly summarized here.

### Patients

FUTURE 2 was conducted at 76 centers throughout Asia, Australia, Europe, and North America, in accordance with the principles of the Declaration of Helsinki, and all centers received approval from independent ethics committees or institutional review boards. Patients included in this trial were aged ≥ 18 years, met the Classification criteria for Psoriatic Arthritis (CASPAR), had ≥ 3 tender and ≥ 3 swollen joints despite treatment with non-steroidal anti-inflammatory drugs (NSAIDs), disease-modifying anti-rheumatic drugs (DMARDs), or TNFis. Patients who had not responded adequately to up to three treatments with TNFis were eligible for inclusion.

A TNFi wash-out period ≥ 4 weeks prior to randomization was mandatory. Patients on concomitant oral corticosteroids who received a stable dose for ≥ 2 weeks prior to randomization (≤ 10 mg/day) and patients who were on methotrexate and received a stable dose for ≥ 4 weeks prior to randomization (≤ 25 mg/week) were allowed throughout the trial. Patients were excluded from the study for previous use of biologic agents other than TNFis; active inflammatory diseases other than PsA; active infection within 2 weeks prior to randomization or a history of chronic, ongoing, or recurrent infections; pregnancy; or malignancy within the past 5 years, with the exception of basal cell carcinoma or actinic keratosis, in-situ cervical cancer, or non-invasive malignant colon polyps.

### Study design and assessments

Patients were randomized 1:1:1:1 to receive subcutaneous secukinumab 300 mg, secukinumab 150 mg, secukinumab 75 mg, or placebo at baseline, weeks 1, 2, and 3, and then every 4 weeks from week 4. At week 16, patients were classified as responders (≥ 20% improvement from baseline in tender and swollen joint counts) or non-responders. Placebo-treated patients were randomly assigned again in a 1:1 ratio to receive subcutaneous secukinumab 300 mg or 150 mg every 4 weeks from week 16 (non-responders) or week 24 (responders). This study was blinded from baseline to week 52 and then open label until week 104.

Pain was assessed through week 104 by the mean change from baseline in the pain VAS and Short Form-36 (SF-36) bodily pain domain scores; proportion of patients reporting improvements equal to or better than the minimum clinically meaningful difference (MCID) in the pain VAS score (mean change from baseline ≥ 20%) [18]; proportion of patients reporting improvements equal to or better than the MCID (5-point improvement from baseline) in SF-36 bodily pain domain score [19]; proportion of patients reporting EuroQoL 5-Dimension 3-Level Questionnaire (EQ-5D-3 L) pain item scores of no, moderate, or extreme pain/discomfort; and correlation coefficients for changes from baseline between pain scores.

### Statistical methods

Mean changes from baseline in the pain VAS and SF-36 bodily pain domain scores were analyzed using a mixed-effect model for repeated measures (MMRM) through week 24 with treatment regimen, analysis visit, and randomization stratum (TNF-naive or TNF-IR) as factors, weight and baseline score as continuous covariates, and treatment by analysis visit and baseline score by analysis visit as interaction terms, and an unstructured covariance structure. Mean changes from baseline were reported as observed starting from week 28. Frequencies of patient EQ-5D-3 L pain/discomfort domain response were reported using observed data up to week 104. Correlation with pain measures was analyzed using Pearson’s correlation coefficient and *P* values were calculated using the chi-square likelihood ratio test. The proportion of patients with VAS improvement ≥ 20% were reported using as-observed data and *P* values were calculated using Fisher’s exact test. The proportion of patients with improvement ≥ 5 in the SF-36 bodily pain domain score were reported using as-observed data and *P* values were calculated using Fisher’s exact test. Pre-specified subgroup analyses (TNF-naïve and TNF-IR) were performed using MMRM through week 24 and data were reported as observed starting from week 28. Only the results for patients receiving the approved doses – secukinumab 300 mg and secukinumab 150 mg – are reported in this article. Due to the re-randomization of patients in the placebo group at week 16, statistical comparisons of secukinumab versus placebo are presented up to week 16. Analyses were exploratory in nature and conducted without adjustment for multiple comparisons.

## Results

### Study population

Overall, 397 patients with active PsA were randomized for this trial. Demographics and baseline disease characteristics have been previously reported for patients in this study and are presented in Additional file 1: Table S1. In the overall patient population, mean pain VAS scores were in the range of 55.4–58.9 and mean SF-36 bodily pain scores were in the range of 33.7–37.6. Further, 99% of patients reported moderate-to-extreme pain or discomfort at baseline measured by the EQ-5D-3 L pain/discomfort item. Of 298 patients, 193 (65%) were TNFi naïve at baseline.

### Efficacy

**Fig. 1:**
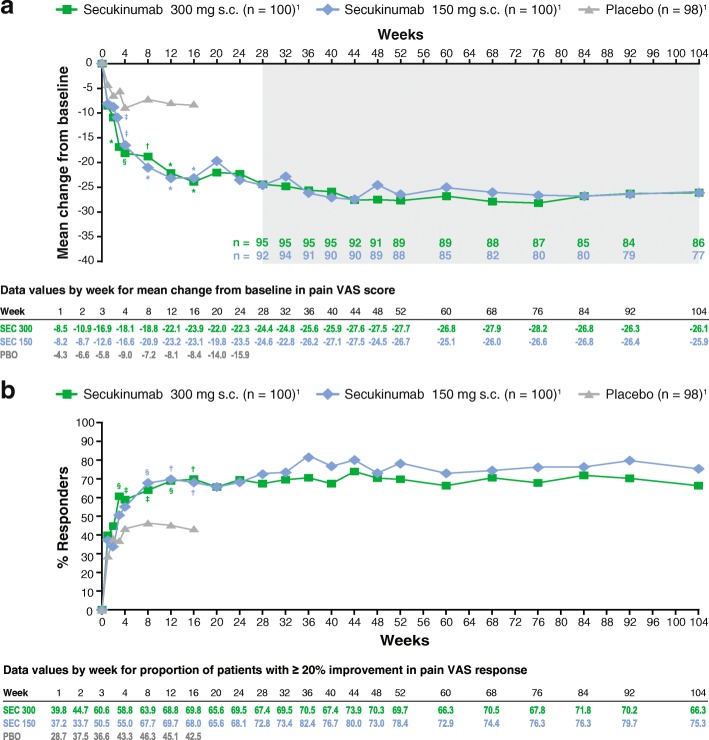
Proportion of patients with ≥ 20% improvement in pain visual analog scale (VAS) score through week 104 (**a**) and proportion of patients with ≥ 20% improvement in pain VAS response (**b**) Data are presented as LS mean change using MMRM from weeks 1-24, and observed data are presented from weeks 28-104 (shaded area); *P* values are calculated from a MMRM analysis (**a**). Observed data are presented; P values are calculated from Fisher’s exact test (**b**) _1_Number of patients originally randomized to each treatment group. *P < 0.0001; †*P* < 0.001; ‡*P* < 0.05 §*P* < 0.01 versus placebo LS, least squares; MMRM, mixed-effect model for repeated measures; PBO, placebo; s.c., subcutaneous; SEC, secukinumab

Treatment with secukinumab was associated with rapid and sustained relief of pain in patients with PsA. Significant improvements in mean changes from baseline in pain VAS scores were reported beginning at week 3 compared with placebo (− 5.8) with secukinumab 300 mg (−16.9; *P* < 0.0001) and secukinumab 150 mg (−12.6; *P* < 0.05). Improvements in mean changes from baseline in pain VAS scores continued to week 16 and were sustained to week 104 with both doses of secukinumab (Fig. 1a). Additionally, most patients treated with secukinumab reported responses equal to or better than the MCID in the pain VAS response. At week 3, a significantly greater number of patients receiving secukinumab 300 mg had ≥ 20% improvement in pain VAS response compared with placebo (60.6% vs 36.6%; *P* < 0.01). For both doses of secukinumab, the proportion of patients with improvements equal to or better than the MCID in pain VAS scores continued to increase to week 16 and similar levels of attainment were reported at week 104 (Fig. 1b).

**Fig. 2:**
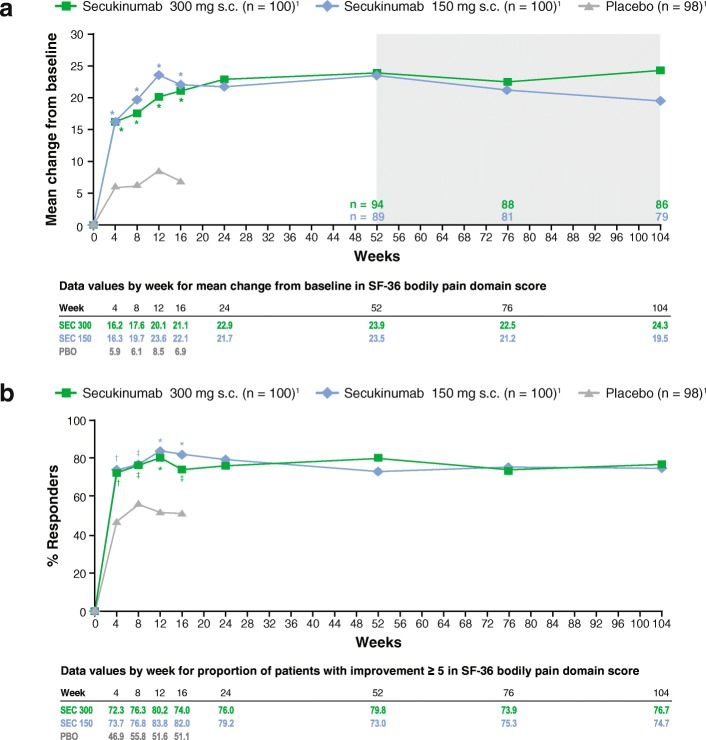
Change from baseline in the Short Form-36 (SF-36) bodily pain score through week 104 (**a**) and proportion of patients with improvement ≥ 5 in the SF-36 bodily pain domain score (**b**) Data are presented as LS mean change using MMRM from weeks 1-24, and observed data are presented from weeks 52-104 (shaded area); *P* values are calculated from a MMRM analysis (**a**). Observed data are presented; *P* values are calculated from Fisher’s exact test (**b**) _1_Number of patients originally randomized to each treatment group. *P < 0.0001; †*P* < 0.001; ‡*P* < 0.05 versus placebo. LS, least squares; MMRM, mixed-effect model for repeated measures; PBO, placebo; s.c., subcutaneous; SEC, secukinumab

A similar trend for reductions in patient-reported pain with secukinumab was reported for SF-36 bodily pain domain scores. At week 4, mean changes from baseline in SF-36 bodily pain scores were significantly greater with both secukinumab 300 mg (16.2) and secukinumab 150 mg (16.3) compared with placebo (5.9; *P* < 0.0001 for both). Continued improvement in SF-36 bodily pain domain scores were reported up to week 12 with both secukinumab doses, and initial improvements were sustained to week 104 for both secukinumab doses (Fig. 2a). Most patients treated with secukinumab also reported equal to or better than the MCID in the SF-36 bodily pain domain score. At week 4, a significantly greater number of patients receiving secukinumab 300 mg (72.3%) and secukinumab 150 mg (73.7%) had improvements ≥ 5 points in SF-36 bodily pain domain score compared with placebo (46.9%; *P* < 0.001 for both). For both doses of secukinumab, the proportion of patients with improvements equal to or better than the MCID in SF-36 bodily pain domain scores were maintained to week 104 (Fig. 2b). The MCID for SF-36 bodily pain domain scores was attained in both anti-TNF-naive and anti-TNF-IR patients receiving secukinumab.

**Fig. 3:**
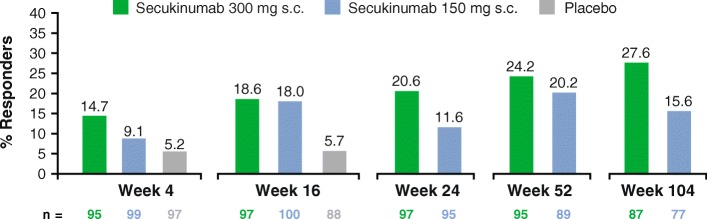
Proportion of patients with no pain or discomfort in the EuroQoL 5-Dimension (EQ-5D-3 L) domain through week 104 Observed data are presented. Data are presented only for evaluable patients at each time point s.c., subcutaneous

At baseline, only 1% of patients reported no pain or discomfort in the EQ-5D-3 L pain/discomfort item. Following 4 weeks of secukinumab treatment, no pain or discomfort was reported by 14.7% of patients receiving secukinumab 300 mg, 9.1% of patients receiving secukinumab 150 mg, and 5.2% of patients receiving placebo (Fig. 3). With secukinumab treatment, the proportion of patients reporting no pain or discomfort continued to increase over time, especially with secukinumab 300 mg and after 2 years of treatment with secukinumab 300 mg almost 30% of patients experienced no pain or discomfort by EQ-5D-3 L.

At weeks 16, 24, 52, and 104, improvements from baseline in mean pain VAS scores were significantly correlated with improvements from baseline in mean SF-36 bodily pain scores (correlation coefficients of − 0.56 to − 0.62; *P* < 0.01 for all) and EQ-5D pain/discomfort domain scores (correlation coefficients of 0.36 to 0.47; *P* < 0.01 for all). Similarly, improvements from baseline in mean SF-36 bodily pain scores were significantly correlated with improvements in EQ-5D pain/discomfort domain scores at weeks 16, 24, 52, and 104 (correlation coefficients of − 0.45 to − 0.52; *P* < 0.01 for all).

### Response by prior TNF inadequate response

TNF-naive and TNF-IR patients both demonstrated significant improvements in mean changes from baseline in pain VAS scores and SF-36 bodily pain scores. TNF-naïve patients receiving secukinumab 300 mg reported significant improvements in mean change from baseline in pain VAS score compared with placebo beginning at week 3 (− 19.7 vs − 8.5; *P* < 0.01). With both doses of secukinumab, improvements continued to week 104 (Table 1). Although a similar trend was observed in TNF-IR patients, the magnitude of improvements was generally lower. In TNF-IR patients, significant mean improvements from baseline in pain VAS scores were reported at week 3 with secukinumab 300 mg (− 13.1; *P* < 0.05 vs placebo) and secukinumab 150 mg (− 14.8; *P* < 0.01 vs placebo). Due to the smaller number of TNF-IR patients there was more volatility in responses over time. Nonetheless, improvements were maintained over time.

**Table 1:** Effect of secukinumab on pain by prior TNF exposure through week 104

	Week 3	Week 4	Week 8	Week 16	Week 24	Week 52	Week 104
*TNF-naïve*							
Pain VAS, mean change from baseline^a^							
Secukinumab 300 mg	−19.7^*^	−20.7^**^	− 20.7^*^	−27.8^****^	− 24.0	−30.3	−29.6
Secukinumab 150 mg	−12.8	−15.2	−21.8^*^	− 25.1^***^	−26.3	−28.1	−28.3
Placebo	−8.5	−11.3	−10.1	−11.3	–	–	–
SF-36 bodily pain, mean change from baseline^b^							
Secukinumab 300 mg	–	18.4^****^	18.2^*^	23.8^****^	23.9	24.4	24.2
Secukinumab 150 mg	–	18.9^****^	20.0^***^	25.4^****^	25.7	25.8	22.2
Placebo	–	5.6	7.3	8.6	–	–	–
EQ-5D-3 L, no pain or discomfort^c^							
Secukinumab 300 mg	–	–	–	21.5%	24.6%	28.6%	32.8%
Secukinumab 150 mg	–	–	–	22.2%	14.8%	20.3%	17.0%
Placebo	–	–	–	6.9%	–	–	–
*TNF-IR*							
Pain VAS, mean change from baseline^d^							
Secukinumab 300 mg	−13.1^**^	−14.9	−16.8^**^	−18.2^**^	−20.7	−22.5	−19.3
Secukinumab 150 mg	−14.8^*^	−21.3^*^	−21.2^*^	−21.1^*^	− 20.0	−23.9	− 20.4
Placebo	−2.1	−5.9	−3.4	−4.4	–	–	–
SF-36 bodily pain, mean change from baseline^e^							
Secukinumab 300 mg	–	15.1	18.7^*^	18.3^*^	23.6	23.0	24.5
Secukinumab 150 mg	–	13.6	21.0^***^	17.9^*^	16.0	19.0	14.0
Placebo	–	7.8	5.7	5.2	–	–	–
EQ-5D-3 L, no pain or discomfort^f^							
Secukinumab 300 mg	–	–	–	12.5%	12.5%	15.6%	17.2%
Secukinumab 150 mg	–	–	–	10.8%	5.9%	20.0%	12.5%
Placebo	–	–	–	3.3%	–	–	–

Least squares mean change using a mixed-effect model for repeated measures (MMRM) from weeks 1 to 24, and observed data from weeks 52 to 104, for pain visual analog scale (VAS) and Short Form-36 (SF-36) bodily pain scores. Observed data are presented for the EuroQoL 5-Dimension 3-Level Questionnaire (EQ-5D-3 L). At week 16, patients initially randomized to placebo who were non-responders switched to secukinumab treatment. Results are not shown for patients who continued on placebo after week 16. Data are presented only for evaluable patients at each time point. TNF-naïve patients originally randomized to secukinumab 300 mg = 67, to secukinumab 150 mg = 63, and to placebo = 63; patients with inadequate response to TNF (TNF-IR) originally randomized to secukinumab 300 mg = 33, to secukinumab 150 mg = 37, and to placebo = 35. *P* values were calculated from a MMRM analysis

^a^Number of evaluable patients, week 52: 59 for secukinumab 300 mg and 150 mg; week 104: 57 for secukinumab 300 mg and 53 for secukinumab 150 mg

^b^Number of evaluable patients, week 52: 62 for secukinumab 300 mg and 59 for secukinumab 150 mg; week 104: 57 for secukinumab 300 mg and 53 for secukinumab 150 mg

^c^Number of evaluable patients, week 52: 63 for secukinumab 300 mg and 59 for secukinumab 150 mg; week 104: 58 for secukinumab 300 mg and 53 for secukinumab 150 mg

^d^Number of evaluable patients, week 52: 30 for secukinumab 300 mg and 29 for secukinumab 150 mg; week 104: 29 for secukinumab 300 mg and 24 for secukinumab 150 mg

^e^Number of evaluable patients, week 52: 32 for secukinumab 300 mg and 30 for secukinumab 150 mg; week 104: 29 for secukinumab 300 mg and 26 for secukinumab 150 mg

^f^Number of evaluable patients, week 52: 32 for secukinumab 300 mg and 30 for secukinumab 150 mg; week 104: 29 for secukinumab 300 mg and 24 for secukinumab 150 mg

^*^P < 0.01; ^**^P < 0.05; ^***^P < 0.001; ^****^P < 0.0001, versus placebo. TNF, tumor necrosis factor

Significant mean improvements from baseline in SF-36 bodily pain scores were also reported by both TNF-naive and TNF-IR patients receiving secukinumab (Table 1). At week 4, mean changes from baseline in SF-36 bodily pain scores in TNF-naïve patients improved by 18.4 with secukinumab 300 mg, by 18.9 with secukinumab 150 mg compared with 5.6 for placebo (*P* < 0.0001 for both versus placebo). Over time, the degrees of improvement in SF-36 bodily pain scores were similar with both doses of secukinumab in TNF-naïve patients. In TNF-IR patients, there were significant mean improvements from baseline in SF-36 bodily pain scores at week 8 with both secukinumab 300 mg (18.7; *P* < 0.01) and 150 mg (21.0; *P* < 0.001) compared to 5.7 with placebo. Mean changes from baseline in SF-36 bodily pain scores were similar over time between TNF-naive and TNF-IR patients with secukinumab 300 mg.

Both TNF-naive and TNF-IR patients also reported improvements in the EQ-5D-3 L pain item scores. At week 16, 22% of TNF-naive patients receiving either dose of secukinumab reported no pain or discomfort compared with 6.9% of patients receiving placebo (Table 1). The proportion of TNF-naive patients receiving secukinumab 300 mg that reported no pain or discomfort continued to increase to week 104 when 32.8% TNF-naive patients had no pain or discomfort. A similar trend was observed in TNF-IR patients but fewer of these patients reported no pain or discomfort compared with TNF-naïve patients. There was a small numerical increase in the number of TNF-IR patients reporting no pain or discomfort at week 104.

## Discussion

Pain is one of the most important domains affecting HRQoL in patients with PsA [7], and patients with PsA report role limitations caused by both emotional problems and bodily pain [5, 20]. Pain has also been linked to fatigue in other inflammatory arthritides [21]. Additionally, joint pain has a quantifiable burden to society as demonstrated by work limitations in patients with PsA [22]. Taken together, these findings exemplify the importance of managing pain when treating patients with PsA.

In this post-hoc analysis of the phase-3, FUTURE 2 study, secukinumab 300 mg and secukinumab 150 mg both provided rapid and sustained pain relief to patients with PsA over 2 years’ treatment. Improvements in pain with secukinumab were consistent across three different patient-reported measures (pain VAS, SF-36 bodily pain, and EQ-5D-3 L pain/discomfort) and were observed regardless of prior exposure to TNFis. Improvements in pain reported in this trial are consistent with findings of improved pain and HRQoL in other secukinumab trials. In FUTURE 1, improvement was observed in both mean pain VAS and SF-36 bodily pain domain scores [19]. Further, secukinumab 150 mg demonstrated sustained improvement in HRQoL with an SF-36 physical component summary score of 4.89 at week 104 [17]. In FUTURE 2, improved physical function was reported at week 24 with a change from baseline in the Health Assessment Questionnaire Disability Index (HAQ-DI) of − 0.56 with secukinumab 300 mg (*P* = 0.004 vs placebo) and − 0.48 with secukinumab 150 mg (*P* = 0.0555 vs placebo) [15].

In this trial, improvements in pain with secukinumab treatment were sufficient to meet or exceed established definitions for MCID in pain for PsA and were observed irrespective of previous exposure to TNFis. Due to the level of improvement in pain with secukinumab treatment, it is interesting to consider whether pain alleviation is due to overall improvement in inflammation or through a direct role of IL-17 inhibition. Plasma levels of IL-17A are elevated in patients with fibromyalgia and in mice, IL-17 contributes to neuroinflammatory responses and pain hypersensitivity following neuropathic injury [23, 24]. These findings suggest that additional investigation is warranted on the role of IL-17 in other conditions associated with pain such as PsA.

There was significant correlation between the improvement from baseline in mean pain VAS scores and mean SF-36 bodily pain scores and EQ-5D pain/discomfort domain scores and significant correlation between improvement from baseline in mean SF-36 bodily pain scores and EQ-5D pain/discomfort scores. Together these correlations indicate that secukinumab consistently improves pain regardless of the specific measurement used for assessment.

Secukinumab 300 mg and secukinumab 150 mg also improved pain in both TNF-naive and TNF-IR patients measured by the pain VAS, SF-36 bodily pain scores, and EQ-5D-3 L pain/discomfort domain. Not surprisingly, TNF-naïve patients reported numerically greater changes from baseline in all three pain outcomes than those reported by TNF-IR patients.

Limitations of this study include that it was a post-hoc analysis, the lack of an active comparator, and lack of placebo control after week 24.

## Conclusions

Secukinumab has previously demonstrated efficacy in treatment of the signs and symptoms of PsA, inhibition of radiographic progression in patients with PsA, and a favorable safety profile [15–17]. In this study, secukinumab provided rapid pain relief for patients with PsA as assessed by multiple clinically relevant patient-reported pain measures. Pain relief began as early as week 3 and improvements were sustained through 104 weeks. Further, improvements in pain were reported by patients regardless of prior exposure to TNFis.

## Additional file

Additional file 1: **Table S1.** Baseline demographics and disease severity characteristics. (DOCX 13 kb)

## Abbreviations

DMARD: Disease-modifying anti-rheumatic drug
EQ-5D-3 L: EuroQoL 5-Dimension 3-Level Questionnaire
EULAR: European League Against Rheumatism
GRAPPA: Group for Research and Assessment of Psoriasis and Psoriatic Arthritis
HRQoL: Health-related quality of life
IL: Interleukin
LOS: Longitudinal observational studies
MAPP: Multinational Assessment of Psoriasis and Psoriatic Arthritis
MCID: Minimum clinically meaningful difference
MMRM: Mixed-effect model for repeated measures
NSAID: Non-steroidal anti-inflammatory drug
OMERACT: Outcome Measures in Rheumatology
PsA: Psoriatic arthritis
PsAID: Psoriatic Arthritis Impact of Disease
RCT: Randomized controlled trial
SF-36: Short Form-36
TNF: Tumor necrosis factor
TNFi: Tumor necrosis factor-α inhibitor
TNF-IR: TNF inadequate response
VAS: Visual analog scale
