# Supplementary material for: Secukinumab provides rapid and sustained pain relief in psoriatic arthritis over 2 years: results from the FUTURE 2 study
Source: Arthritis Res Ther. 2018 Jun 7;20:113. doi: 10.1186/s13075-018-1610-3 (PMC5992664; doi:10.1186/s13075-018-1610-3)
Supplement: Supplementary file 1 — Table S1. Baseline demographics and disease severity characteristics. (DOCX 13 kb) [file 13075_2018_1610_MOESM1_ESM.docx]

**Table S1.** Baseline demographics and disease severity characteristics

| Characteristic | Secukinumab  300 mg s.c.  (*n* = 100) | Secukinumab  150 mg s.c.  (*n* = 100) | Placebo  (*n* = 98) |
| --- | --- | --- | --- |
| Age (years) | 46.9 (12.6) | 46.5 (11.7) | 49.9 (12.5) |
| Female, n (%) | 49 (49.0) | 45 (45.0) | 59 (60.2) |
| Weight (kg) | 85.4 (18.4) | 91.2 (19.8) | 86.2 (19.8) |
| Caucasian, n (%) | 96 (96.0) | 90 (90.0) | 94 (95.9) |
| Anti-TNF-naïve, n (%) | 67 (67.0) | 63 (63.0) | 63 (64.3) |
| Physician’s global assessment (VAS) score | 55.0 (14.7) | 56.7 (16.6) | 55.0 (16.0) |
| Pain (VAS) score | 57.7 (19.0) | 58.9 (19.8) | 55.4 (22.1) |
| EQ-5D | 51.9 (19.4) | 48.5 (20.4) | 52.1 (20.6) |
| EQ-5D Pain/discomfort |  |  |  |
| No pain/discomfort, n (%) | 1 (1.0)* | 1 (1.0) | 1 (1.0) |
| SF-36 PCS | 36.9 (8.0) | 36.2 (8.1) | 37.4 (8.8) |
| SF-36 bodily pain | 35.9 (19.0)* | 33.7 (16.3) | 37.6 (18.8) |
| Patient’s global assessment (VAS) score | 60.7 (18.9) | 62.0 (19.5) | 57.6 (19.8) |
| Dactylitis, n (%) | 46 (46.0) | 32 (32.0) | 27 (27.6) |
| Enthesitis, n (%) | 56 (56.0) | 64 (64.0) | 65 (66.3) |
| TJC (78 joints) | 20.2 (13.3) | 24.1 (19.4) | 23.4 (19.0) |
| SJC (76 joints) | 11.2 (7.8) | 11.9 (10.1) | 12.1 (10.7) |
| DAS28-CRP | 4.8 (1.0) | 4.9 (1.1) | 4.7 (1.1) |
| HAQ-DI | 1.3 (0.6) | 1.2 (0.6) | 1.2 (0.7) |

Data are mean (SD), unless otherwise noted.

Data assessed based on evaluable patients at Baseline.

*There were 99 evaluable patients at baseline for EQ-5D Pain/discomfort and SF-36 bodily pain.

DAS28-CRP, Disease Activity Score 28-C reactive protein; EQ-5D, EuroQoL 5-Dimension; HAQ-DI, Health Assessment Questionnaire-Disability Index; PCS, physical component summary; s.c., subcutaneous; SF-36, Short Form-36; SJC, swollen joint count; TJC, tender joint count; TNF, tumor necrosis factor; VAS, visual analog scale.
